# Suppression of epidemic spreading in complex networks by local information based behavioral responses

**DOI:** 10.1063/1.4896333

**Published:** 2014-10-20

**Authors:** Hai-Feng Zhang, Jia-Rong Xie, Ming Tang, Ying-Cheng Lai

**Affiliations:** 1 School of Mathematical Science, Anhui University, Hefei 230039, China; 2 School of Electrical, Computer and Energy Engineering, Arizona State University, Tempe, Arizona 85287, USA; 3 Department of Communication Engineering, North University of China, Taiyuan, Shan'xi 030051, People's Republic of China; 4 Department of Modern Physics, University of Science and Technology of China, Hefei 230026, China; 5 Web Sciences Center, University of Electronic Science and Technology of China, Chengdu 611731, China

## Abstract

The interplay between individual behaviors and epidemic dynamics in complex networks is a topic of recent interest. In particular, individuals can obtain different types of information about the disease and respond by altering their behaviors, and this can affect the spreading dynamics, possibly in a significant way. We propose a model where individuals' behavioral response is based on a generic type of local information, i.e., the number of neighbors that has been infected with the disease. Mathematically, the response can be characterized by a reduction in the transmission rate by a factor that depends on the number of infected neighbors. Utilizing the standard susceptible-infected-susceptible and susceptible-infected-recovery dynamical models for epidemic spreading, we derive a theoretical formula for the epidemic threshold and provide numerical verification. Our analysis lays on a solid quantitative footing the intuition that individual behavioral response can in general suppress epidemic spreading. Furthermore, we find that the hub nodes play the role of “double-edged sword” in that they can either suppress or promote outbreak, depending on their responses to the epidemic, providing additional support for the idea that these nodes are key to controlling epidemic spreading in complex networks.

Outbreaks of epidemics can trigger spontaneous behavioral responses of individuals to take preventive measures, which in turn can alter the epidemic dynamics and affect the disease transmission process. To study the interplay between behavioral response and epidemic spreading has attracted recent attention. In spite of the efforts, a quantitative picture taking into consideration physical reality of the epidemic dynamical process is needed. Here, we propose a model in which individuals' behavioral responses are based on a generic type of local information, i.e., the number of neighbors that have been infected with the disease. This should be contrasted with existing works in which the responses are based on the density of infection among the local neighborhood or on global information. Utilizing the standard SIS (susceptible-infected-susceptible) and SIR (susceptible-infected-refractory) dynamical processes for modeling epidemic spreading, we derive theoretical formulas for the epidemic thresholds and provide numerical verification. Our main finding is that individual behavioral response can in general augment significantly the epidemic threshold, thereby suppressing the prevalence of epidemic effectively, regardless of type of the dynamics. Especially, the hub nodes in the network can adaptively and actively generate cautious responses to protect themselves and hence many other nodes in the network. The hub nodes thus play the role of “double-edged sword” in epidemic dynamics as they can either suppress or promote outbreak. Our work reinforces the idea that hub nodes are key to controlling epidemic dynamics.

## I. INTRODUCTION

Epidemic spreading in complex networks often occurs in an extremely interactive manner. Consider, for example, the spread of a virus. When individuals become aware of the potential disease, they would take preventive measures (e.g., using better hygiene, wearing protective masks, or avoiding congested public places) to protect themselves and those around them. In this sense, individuals in the network cannot be treated as “passive” nodes awaiting to be infected but they can in fact be quite “reactive” to the spreading dynamics. Such human behavioral responses can have significant effects on the epidemic dynamics,^1–5^ a topic of great recent interest.^6–14^

The individual reactions to an epidemic often rely on detailed information about the disease. Broadly, there are two types of information: local or global.^15^ For example, news obtained from the social neighborhood of an individual is local, but information from the mass media or from the public health authorities can be regarded as global. The influences of local^16–20^ or global^21–23^ information based behavioral responses, or awareness, on the epidemic dynamics can in general be quite different, and there is also recent work on the effects of combined local and global information based awareness.^24–27^ Quantitatively, the impact of different types of information-based awareness can be characterized by how they modify the epidemic threshold and the final epidemic size (or epidemic prevalence). For example, Bagnoli *et al.*^26^ assumed that individuals' risk perception of epidemic is an exponential function of local and global information, and they showed that a nonlinear increase in the perception risk can lead to extinction of the disease. Funk *et al.*^17^ studied the impacts of awareness spread on both epidemic threshold and prevalence and found that, in a well-mixed population, spread of awareness can reduce the outbreak size but does not tend to affect the epidemic threshold. This result, however, does not appear to hold for social networks for which the mixed-population assumption is not valid. In particular, Wu *et al.*^24^ compared the roles of the three forms of information-based awareness, i.e., local, global, and contact awareness, in the epidemic threshold, and concluded that global awareness cannot alter the epidemic threshold, while both local and contact awareness can. Sahneh *et al.*^6,7^ proved that local information-based response can enhance the epidemic threshold and reduce the prevalence, given that the probability of susceptible individuals to alter their state is proportional to the number of the infected neighbors.

Our work is motivated by the following two considerations. First, a general result from previous works is that the local information-based responses can enhance the epidemic threshold and reduce its prevalence, but global information-based awareness, although being capable of altering the epidemic size, has little effect on the threshold.^6,24,25^ In these works on the interplay between epidemic spreading in complex networks and human behavioral responses, a tacit hypothesis^24–26,28^ is that local information-based behavioral response is a function of the density of infection among the local neighborhood, denoted as *s*/*k*, where *s* is the number of infected neighbors among a total of *k* neighbors. However, simple situations can be conceived where this assumption does not hold. For example, consider two nodes in a complex network, *i* and *j*, which have 10 and 100 neighbors, respectively. Assume that in their respective neighborhoods, there are 5 and 50 infected nodes. The hypothesis would then assign the two nodes with the same value of *s*/*k*, or identical risk perception. However, common sense stipulates that node *j*, because of the much larger number of infected neighbors, should have stronger awareness about the epidemic than node *i*. A real-world example is some popular websites or important network routers that have a large probability of being attacked by virus. As a result, for various reasons they tend to be much better protected.

The second motivation of our work is that most recent works addressing the roles of individual behavioral response tend to focus on one type of epidemic process, e.g., either SIS or SIR dynamics. A key difference between SIS and SIR dynamics is that the former is reversible while the latter is irreversible.^29,30^ Another difference is that, for SIS dynamics, the system can only reach a *dynamically* steady state since the propagation process always occurs once the transmission rate exceeds the epidemic threshold. However, for the SIR process, propagation will terminate once there are no longer infected nodes in the network. These differences can lead to different interplay between the epidemic dynamics and behavioral response, demanding a systematic comparison study.

In this paper, we introduce a realistic local information-based behavioral response mechanism into both SIS and SIR dynamics. In particular, we assume that individuals generate behavioral responses by reducing their contact rates, depending on *the actual number of infected neighbors* (not the density of such neighbors), and study how the epidemic thresholds and prevalence for both types of epidemic dynamics are altered. We find that the mechanism generates similar effects on the SIS and SIR epidemic thresholds but lead to different epidemic sizes for the two types of dynamics. An important consequence of our realistic behavioral response mechanism is that, for both SIS and SIR dynamics, the infection densities can be maximized for some intermediate values of the node degree. This implies that both small- and large-degree nodes are relatively more resilient to infection, in sharp contrast to the monotonic increase of the infection density with the node degree as in situations where no behavioral response is taken into account.^31,32^

In Sec. II, we describe our model of epidemic spreading with local information based behavioral response. In Sec. III, we develop theoretical analyses with numerical support to understand the effects of such response on the spreading dynamics in terms of the two fundamental quantities: epidemic threshold and prevalence. Due to the intrinsic difference between SIS and SIR processes, we shall treat them using different theoretical methods. In Sec. IV, we present conclusion and discussions.

## II. MODELING BEHAVIORAL RESPONSE

Epidemic spreading is a fundamental type of network dynamical process that has been studied extensively due to the development of complex networks. In a network, a node represents an individual and an edge between a pair of nodes specifies a contact through which the epidemic can diffuse or propagate.^33–37^ In the absence of any behavioral response, for an unweighted network the contact rate of every pair of nodes can be conveniently set to be unity. A reasonable assumption is that an individual would become more cautious and therefore take more effective preventive measure if many of the neighbors have been infected. The behavioral response can then be modeled quantitatively by introducing the reduction factor (1 – *α*)^*s*^ in the contact rate of a susceptible node, where *s* is the number of infected neighbors and 0 ≤ *α* < 1 is a parameter characterizing the response strength of the individuals to the epidemic. The larger the value of *α*, the more cautious the individual becomes, resulting a more substantial reduction in the contact rate. In a real situation, the value of *α* would vary across all the individuals in the network. Here, for simplicity and for gaining insights into the epidemic dynamics subject to behavioral response, we assume identical value of *α* for all nodes in the network. For the trivial case of *α* = 1.0, the contact rate will *immediately* become zero once a neighbor is infected, ruling out any epidemic outbreak. Let *λ* be the original transmission rate along each edge. The new transmission rate in our model is $\tilde{\lambda }=\lambda {\left(1-\alpha \right)}^{s}$.

Our goal is to investigate, quantitatively, the effect of reduced contact rates due to the individual behavioral response on the epidemic dynamics in terms of the two basic quantities: epidemic threshold and prevalence. In the following, we will treat the SIS and SIR processes separately.

## III. EFFECT OF BEHAVIORAL RESPONSE ON EPIDEMICS: THEORY AND NUMERICAL VALIDATION

### A. SIS dynamics

We first consider the standard SIS epidemic model on a network with general degree distribution *P*(*k*). At any time, each node in the network can be in one of the two states: susceptible (S) or infected (I). The transmission probability along each SI edge is $\tilde{\lambda }$. With probability *μ*, an infected individual recovers and returns to the susceptible state. For convenience,^38^ we set *μ* = 1.0.

We use the standard degree-based approximation (heterogeneous mean-field approximation)^39–42^ to analyze the epidemic dynamics, in which all nodes of the same degree are assumed to have the same probability of infection at any given time. In particular, letting Θ(*t*) be the probability that a randomly selected edge points to an infected individual at time *t*, we have

$$\Theta (t)=\sum_{k}Q(k-1)\rho_{k}(t)=\frac{1}{〈k〉}\sum_{k}kP(k)\rho_{k}(t),$$ (1)

where *ρ_k_*(*t*) is the probability that a node with degree *k* is infected and Q(k−1)=kP(k)/〈k〉 is the excess degree distribution in the absence of degree-to-degree correlation.^43^ The probability that a node with degree *k* has exactly *s* infected neighbors is then given by^24^

$$B(k,s)={(}_{s}^{k})\Theta^{s}{\left(1-\Theta \right)}^{k-s}.$$ (2)

For a susceptible node with *k* neighbors, among which *s* are infected, in a sufficiently small time interval [*t*, *t* + Δ*t*] (Δ*t* → 0) the probability of infection is

$$\varphi (s)=1-{\left[1-\Delta t\lambda {\left(1-\alpha \right)}^{s}\right]}^{s}≃\lambda s{\left(1-\alpha \right)}^{s}\Delta t.$$ (3)

The average probability that a susceptible node with *k* neighbors is infected is

$$\begin{array}{c} \text{Prob}(S\to I)≅E[\varphi (s)]=\sum_{s=0}^{k}B(k,s)\varphi (s)\\ =\lambda \Delta t\sum_{s=0}^{k}B(k,s)s{\left(1-\alpha \right)}^{s}\\ =\lambda \Delta t(1-\alpha )k\Theta \sum_{s=1}^{k}{(}_{s-1}^{k-1}){\left[\Theta (1-\alpha )\right]}^{s-1}{\left(1-\Theta \right)}^{\left(k-s\right)}\\ =\lambda \Delta t(1-\alpha )k\Theta {\left(1-\alpha \Theta \right)}^{k-1}. \end{array}$$ (4)

According to Eq. (4), the discrete-time epidemic process can be described as

$$\begin{array}{c} \rho_{k}(t+\Delta t)-\rho_{k}(t)\\ =-\Delta t\rho_{k}(t)+(1-\rho_{k}(t))\lambda \Delta t(1-\alpha )k\Theta {\left(1-\alpha \Theta \right)}^{k-1}. \end{array}$$ (5)

In the limit Δ*t* → 0, Eq. (5) can be written as a continuous-time equation

$$\frac{d\rho_{k}(t)}{dt}=-\rho_{k}(t)+(1-\rho_{k}(t))\lambda (1-\alpha )k\Theta {\left(1-\alpha \Theta \right)}^{k-1}.$$ (6)

For *α* = 0, Eq. (6) reduces to the standard mean-field equation for the SIS model.^34,44^

Imposing the steady-state condition *dρ_k_*(*t*)/*dt* = 0 on Eq. (6), we obtain

$$\rho_{k}=\frac{\lambda (1-\alpha )k\Theta {\left(1-\alpha \Theta \right)}^{k-1}}{1+\lambda (1-\alpha )k\Theta {\left(1-\alpha \Theta \right)}^{k-1}}.$$ (7)

Substituting Eq. (7) into Eq. (1), we obtain the following self-consistent equation:

$$\begin{array}{c} \Theta (t)=\frac{\lambda \Theta (1-\alpha )}{〈k〉}\sum_{k}\frac{P(k)k^{2}{\left(1-\alpha \Theta \right)}^{k-1}}{1+\lambda k\Theta (1-\alpha ){\left(1-\alpha \Theta \right)}^{k-1}}\\ =f(\Theta ). \end{array}$$ (8)

A nonzero steady infection size is obtained when the following inequality holds:

$$\frac{df(\Theta )}{d\Theta }{|}_{\Theta =0}\geq 1,$$ (9)

which gives the epidemic threshold as

$${\tilde{\lambda }}_{c}^{SIS}=\frac{1}{\left(1-\alpha \right)}\frac{〈k〉}{〈k^{2}〉}=\frac{\lambda_{c}^{SIS}}{1-\alpha },$$ (10)

where $\lambda_{c}^{SIS}=〈k〉/〈k^{2}〉$ is the epidemic threshold for the classical SIS model in heterogeneous networks^34,44^ and $〈k^{2}〉=\sum_{k}k^{2}P(k)$ is the second moment of the degree distribution.

To validate our analysis, we consider networks generated from the standard configuration model^45^ with degree distribution *P*(*k*) ∼ *k*^−3^. The size of all networks studied is *N* = 10 000, the minimal and maximal degrees are *k_min_* = 3 and $k_{max}=\sqrt{N}=100$, respectively. The results presented below are insensitive to network structural and/or parameter changes.

**FIG. 1.**
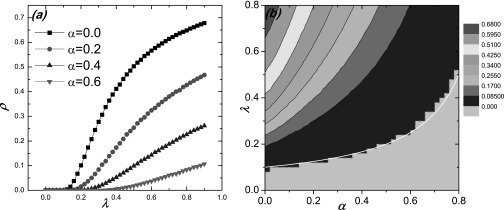
Effect of behavioral response strength *α* on the epidemic threshold and prevalence for SIS model: (a) final epidemic size *ρ* versus the transmission rate *λ* for different values of *α*; (b) contour of *ρ* in the (*λ* – *α*) parameter plane, where the white line denotes the theoretically predicted curve associated with the epidemic threshold [Eq. (10)], and the light gray region divided by the dash pink line corresponds to the zero prevalence. The number of the initial infected seeds is *I*_0_ = 5. Each point is the statistical average of 20 random network configurations and 50 independent initial conditions for each network realization.

Figure 1 illustrates how the transmission rate *λ* and the behavioral-response strength *α* affect the epidemic prevalence and threshold. In particular, from Fig. 1(a), we see that the epidemic threshold increases with *α* but the final epidemic size (prevalence) rapidly decreases to a low value. Figure 1(b) shows the numerically calculated contours of the values of epidemic prevalence in the parameter plane (*λ* – *α*), with the theoretically predicted curve (white line) for the epidemic threshold (corresponding to zero prevalence). We observe a good agreement between theory and numerics. For relatively large values of *α*, the lowest line in Fig. 1(b) deviates somewhat from the theoretical value with small oscillations. The reason lies in the difficulty to numerically distinguish the two cases where the epidemic is suppressed or prevalent for the large *α* regime.

**FIG. 2.**
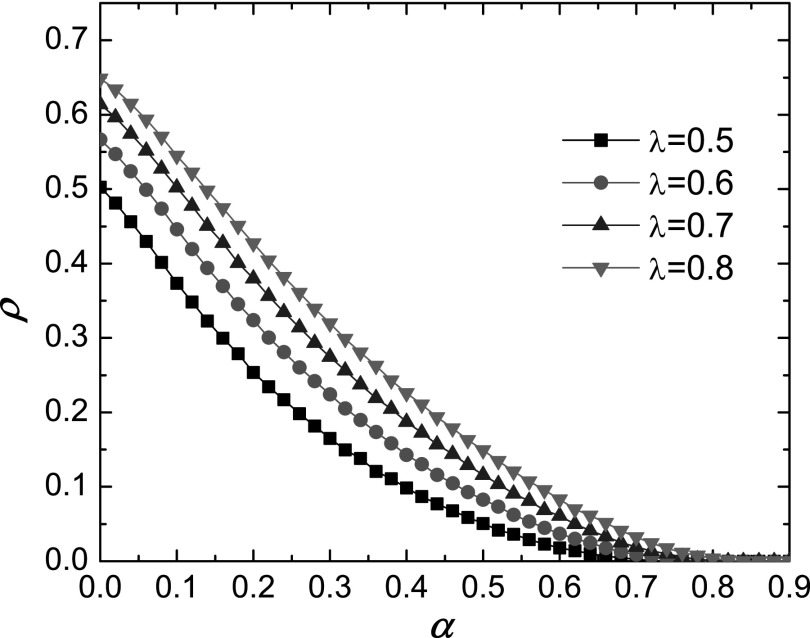
For the same parameter setting as in Fig. 1, simulation shows the dependence of the epidemic prevalence *ρ* on *α*.

To further reveal the impact of behavioral response on the epidemic process, we show in Fig. 2 the dependence of the epidemic prevalence *ρ* on *α* for different values of *λ*, where we observe a rapid decrease (faster than linear) in *ρ* as *α* is increased. This indicates that proper behavioral response can greatly suppress epidemic outbreak. For example, for *α* ≈ 0.8, epidemic is nearly eliminated.

**FIG. 3.**
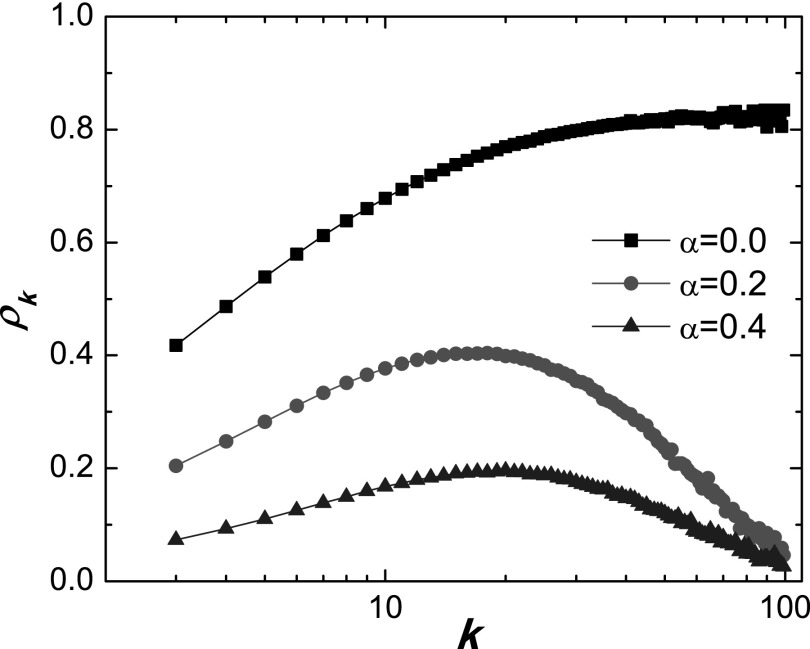
For SIS dynamics on scale-free networks, degree-dependent steady-state infection density *ρ_k_* for different values of *α*. The original transmission rate is *λ* = 0.5 and other parameters are the same as for Fig. 1.

For the classical SIS dynamics on heterogeneous networks, it has been established that the degree-specific infection density *ρ_k_* increases with *k*, implying that hub nodes should have a higher probability of being infected.^34,44^ However, when behavioral response is taken into account, the situation becomes somewhat complicated. For example, a hub node would possibly have many infected neighbors, making it better informed about the epidemic and consequently generating a stronger protective response. Figure 3 shows how *ρ_k_* depends on the degree for different values of *α*. For *α* = 0 (the standard SIS model without any behavioral response), *ρ_k_* increases with degree *k*. However, for *α* > 0, e.g., *α* = 0.2 and *α* = 0.5, we obtain a non-monotonic dependence: *ρ_k_* first increases with *k*, reaches a maximal value, and then begins to decrease with *k* and approach zero for very large values of *k*. This case can be quantitatively explained using Eq. (7) where, for a fix value of Θ, the value of (1 – *α*Θ)^*k*^ converges to zero in the numerator. As a result, we have *ρ_k_* = 0 for sufficiently large values of *k*. The increasing phase can be understood by noting that nodes with small degree have lower probabilities to reduce their contact rate because they are typically unaware of the disease as their neighbors are few and the infected neighbors are even fewer. As *k* is increased from some low value, the probability of infection is increased (as for the standard SIS dynamics without behavioral response).

We thus see that, in our model, the hub nodes are capable of inhibiting epidemic spreading, providing an effective way to control the dynamics. This should be contrasted to the traditional models that do not take into account individuals' behavioral responses in which the role of the hub nodes is generally understood as enhancing epidemic spreading.

In general, there are two competing factors determining the epidemic size: probability of infection and information from the neighbors. In the small-degree regime, the former has a stronger effect, leading to an increase in the epidemic prevalence with the degree. In the intermediate degree regime, the effects of the two factors are approximately balanced, so the final epidemic size reaches a maximum. Finally, in the large degree regime, the effect of awareness and hence behavioral response exceeds that of the infection, leading to a significant reduction in the transmission rate and consequently a continuous decrease in the epidemic prevalence with the degree. The same mechanism explains the rapid decrease in the epidemic prevalence with *α*.

### B. SIR dynamics

In SIR dynamics, infected nodes can enter a recovery state (R) to become immune to the disease. The underlying epidemic process is then an irreversible process, as a result, we should provide a modification in the way to characterize behavioral response for this case. Specifically, we assume that susceptible nodes adjust the transmission rate based not only on the number of infected nodes but also on the number of *recovered* nodes. This is reasonable as any recovered neighbor of a node has already gone through the infection stage and therefore is able to inform the node about the disease. To analyze the resulting SIR dynamics, we find the generating function method and cavity theory^30,46^ suitable for calculating the critical epidemic threshold.

We first define “externally infected neighbor” (EIN) for any node.^47^ For node *i*, if a neighbor is an EIN, it is infected by its neighbors other than *i*. We then define *u* to be the probability that *i*'s neighbor *j* is an EIN of *i*, i.e., the probability that node *j* is infected even when *i* is removed from the network (the basic assumption of the cavity theory in statistical physics^47^). The probability that a node with degree *k* has *m* EINs is then

$$p(m|k)={(}_{m}^{k})u^{m}{\left(1-u\right)}^{k-m}.$$ (11)

Let $\tilde{p}(R|m)$ be the conditional probability of infection if one node *i* has *m* EINs. The EINs typically appear in a sequential order, so we need to calculate $\tilde{p}(R|m)$ in a time-ordered fashion. In particular, when the first EIN appears, the probability of node *i* not being infected is 1 – *T*_1_, where *T*_1_ ≡ *λ*(1 – *α*) is the probability that *i* is infected after the first EIN appears in its neighborhood. In general, the probability that node *i* has not been infected after *m*th EINs appear in its neighborhood is 1 – *T_m_*, where *T_m_* = *λ*(1 – *α*)^*m*^. We thus have

$$\tilde{p}(R|m)=1-(1-T_{1})(1-T_{2})\cdots (1-T_{m}).$$ (12)

In general, it is difficult to simplify the expression of $\tilde{p}(R|m)$. However, if we focus on the epidemic threshold, the probability *T_m_* will be small since the corresponding value of *λ* [or (1 – *α*)] is small. In this case, Eq. (12) can be approximated as

$$\tilde{p}(R|m)≃\sum_{j=1}^{m}T_{j}=\frac{\lambda (1-\alpha )}{\alpha }[1-{\left(1-\alpha \right)}^{m}].$$ (13)

For a randomly selected node *i*, the probability of having *m* EINs is

$$p(m)=\sum_{k=m}^{\infty }p(m|k)P(k).$$ (14)

For a randomly selected neighbor *j*, the probability that it has exactly *m* EINs (excluding *i*) is

$$q(m)=\sum_{k=m}^{\infty }p(m|k)Q(k),$$ (15)

where Q(k)=(k+1)P(k+1)/〈k〉 is the excess degree distribution.^43^ The generating function associated with the excess-degree distribution is

$$G_{1}(x)=\sum_{k}Q(k)x^{k}.$$ (16)

Combining Eqs. (11), (15), and (16), we obtain the generating function for the probability *q*(*m*) as

$$\begin{array}{c} F_{1}(x)=\sum_{m}q(m)x^{m}=\sum_{m=0}^{\infty }\sum_{k=m}^{\infty }p(m|k)Q(k)x^{m}\\ =\sum_{k\geq m\geq 0}p(m|k)Q(k)x^{m}\\ =\sum_{k=0}^{\infty }Q(k)\sum_{m=0}^{k}{(}_{m}^{k})u^{m}{\left(1-u\right)}^{k-m}x^{m}\\ =\sum_{k=0}^{\infty }Q(k){\left(1-u+xu\right)}^{k}\\ =G_{1}(1-u+xu). \end{array}$$ (17)

From the definition of the probability *u*, we have

$$\begin{array}{c} u=\sum_{m}q(m)\tilde{p}(R|m)=\frac{\lambda (1-\alpha )}{\alpha }\sum_{m}q(m)[1-{\left(1-\alpha \right)}^{m}]\\ =\frac{\lambda (1-\alpha )}{\alpha }[1-F_{1}(1-\alpha )]\\ =\frac{\lambda (1-\alpha )}{\alpha }[1-G_{1}(1-\alpha u)]=f(u), \end{array}$$ (18)

which is a self-consistent equation for *u*. There is a trivial solution *u* = 0. In order to have a non-trivial solution, the following condition must be met:

$$\frac{df(u)}{du}{|}_{u=0}=\frac{\lambda (1-\alpha )}{\alpha }[\alpha G\prime_{1}(1)]\geq 1,$$ (19)

which implies

$$\lambda \geq {\tilde{\lambda }}_{c}^{SIR}=\frac{1}{1-\alpha }\frac{〈k〉}{〈k^{2}〉-〈k〉}=\frac{1}{1-\alpha }\lambda_{c}^{SIR},$$ (20)

where $\lambda_{c}^{SIR}=〈k〉/(〈k^{2}〉-〈k〉)$ is the epidemic threshold of SIR dynamics on heterogeneous networks in the absence of any behavioral response.^30^

**FIG. 4.**
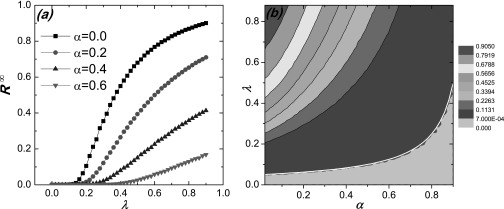
For SIR dynamics, effect of local behavioral response on epidemic prevalence and threshold: (a) epidemic prevalence *R^∞^* as a function of transmission rate *λ* for different values of *α*; (b) contour of *R^∞^* with respect to *λ* and *α*, where the white line is the prediction of Eq. (20). The gray region under the pink dash link indicates the epidemic prevalence $R^{\infty }<7\times 10^{-4}$. Since the number of the initial infected seeds is *I*_0_ = 5, we have $R^{\infty }\geq 5/N=5\times 10^{-4}$ for the steady state. Other parameters are the same as for Fig. 1.

Figure 4 shows the dependence of the SIR epidemic prevalence [panel (a)] and threshold [panel (b)] on *λ* and *α*. Fig. 4(a) indicates that the epidemic prevalence *R^∞^* decreases with *α* but the critical threshold ${\tilde{\lambda }}_{c}^{SIR}$ increases with *α*. Figure 4(b) presents the theoretical prediction from Eq. (20) (white line) in comparison with the simulation result for near-zero *R^∞^* value. We observe a good agreement.

**FIG. 5.**
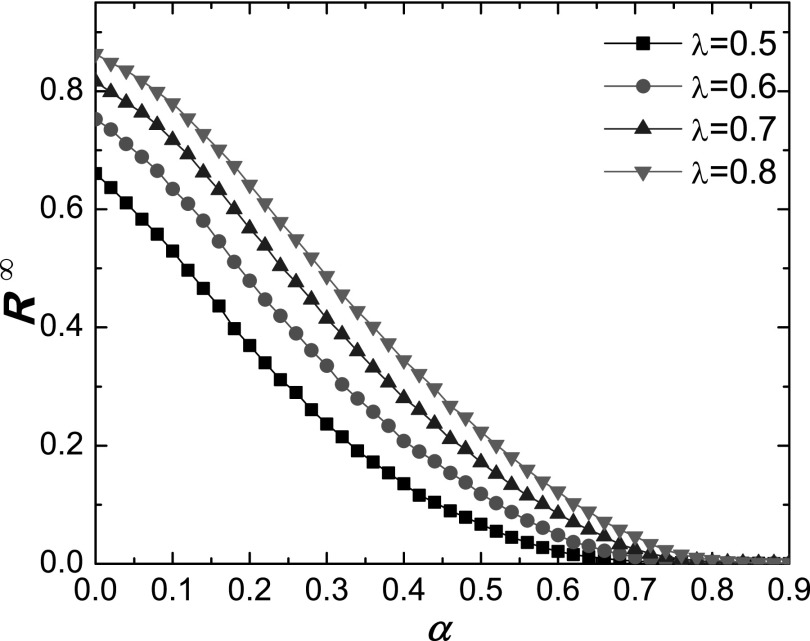
For SIR dynamics, simulation shows the epidemic prevalence *R^∞^* versus *α* for different values of *λ*. Other parameters are the same as for Fig. 1.

**FIG. 6.**
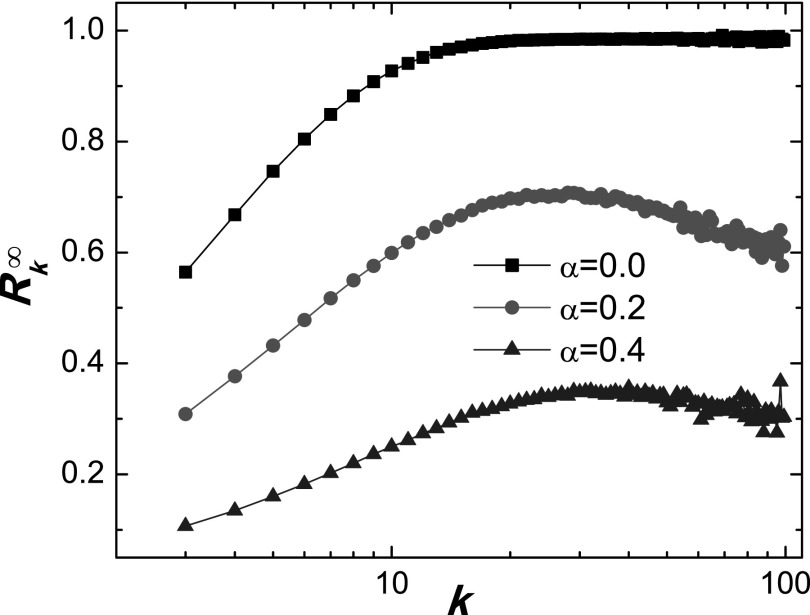
For SIR dynamics, final recovery density $R_{k}^{\infty }$ versus degree *k* for different values of *α*, for *λ* = 0.5. Other parameters are the same as for Fig. 1.

Figure 5 shows the dependence of *R^∞^* on *λ* for different values of *α*. Comparing Fig. 2 with Fig. 5, we can see that, for SIR dynamics with local behavioral response, the epidemic prevalence also rapidly decreases with *α*, demonstrating that such a response can be effective to suppress epidemic spreading, as for the case of SIS dynamics. Since, for an SIS process, the system can sustain a dynamically steady state in which the infected individuals are more likely to have their contact rates reduced after becoming susceptible again,^48^ one might intuitively expect the effect of behavioral response to be somewhat different. In particular, the intrinsically irreversible SIR dynamics can cause the system to quickly converge to a steady state. As a result, the hub nodes are infected relatively soon and they have no chance to make any behavioral responses, limiting their role in suppressing the spreading. However, we note that the susceptible nodes can adjust their transmission rates based not only on the number of infected nodes but also on the number of recovered nodes, so as to greatly enhance the awareness of hub nodes and effectively reducing the probability of their being infected. Support for this heuristic reasoning can be found in Fig. 6, where the final recovery density $R_{k}^{\infty }$ as a function of degree *k* for the SIR model is shown. We see that, even though the hub nodes cannot be completely protected from the epidemic, $R_{k}^{\infty }$ reduces to a low level and decreases as *α* is increased. Consequently, local behavioral response associated with SIR dynamics can also be effective in suppressing epidemic spreading.

## IV. CONCLUSIONS

Recognizing that individuals in a social network have the natural tendency to respond to potential disease spreading by taking preventive measures, we investigated to what extent behavioral responses based on *local* information can affect typical epidemic dynamics. Using two standard types of processes in epidemiology, SIS and SIR, as prototypical dynamical model, we developed theoretical analysis to understand how the two fundamental characterizing quantities, the epidemic threshold and prevalence, are shaped by local-information based behavioral response. We found that such response can in general augment significantly the epidemic threshold, regardless of SIS or SIR dynamics, and we obtained an explicit expression for the augmentation factor. This means that individual behavioral responses can make the whole network much more resilient to epidemic outbreak. In the case where outbreak has occurred, the final epidemic size, or epidemic prevalence, can be reduced.

A unique feature of our work is the assumption that individual response is determined by the local information, which in turn is proportional to the number of infected neighbors. Our findings complement the previous results in the absence of any individual response that epidemic can prevail in heterogeneous networks due to the existence of hub nodes. Particularly, when individuals are able to receive information about the disease and are capable of taking preventive measures, it is the hub nodes that can adaptively and actively generate responses to protect themselves and hence many other nodes in the network. The hub nodes thus play the role of “double-edged sword” in epidemic dynamics as they can either suppress or promote outbreak, depending on how they respond to epidemic. Our work reinforces the idea that hub nodes are key to controlling epidemic dynamics.

We focused on an epidemic response model where the individuals respond to the epidemic according to the number of infected neighbors. There are real world situations where this is meaningful. For example, a node with potentially a large number of infected neighboring nodes would have stronger awareness about the epidemic. Our model thus complements the previous model in which the local information-based behavioral response is assumed to be a function of the density of infected nodes in the local neighborhood.
